# Individualized C1-2 intra-articular three-dimensional printed porous titanium alloy cage for craniovertebral deformity

**DOI:** 10.1186/s13018-024-05049-4

**Published:** 2024-09-16

**Authors:** Qiang Jian, Shaw Qin, Zhe Hou, Xingang Zhao, Cong Liang, Tao Fan

**Affiliations:** 1 Spine Center, Sanbo Brain Hospital, Capital Medical University, Beijing, People’s Republic of China; 2 Department of Neurological Surgery, Columbia University, The Och Spine Hospital at NewYork-Presbyterian, New York, NY USA

**Keywords:** 3D printing, Intra-articular cage, Craniovertebral deformity, CVJ, Basilar invagination, Atlantoaxial instability

## Abstract

**Background:**

Congenital craniovertebral deformity, including basilar invagination (BI) and atlantoaxial instability (AAI), are often associated with three-dimensional (3D) deformity, such as C1-2 rotational deformity, craniocervical kyphosis, C1 lateral inclination, among other abnormalities. Effective management of these conditions requires the restoration of the 3D alignment to achieve optimal reduction. Recently, 3D printing technology has emerged as a valuable tool in spine surgery, offering the significant advantage of allowing surgeons to customize the prosthesis design. This innovation provides an ideal solution for precise 3D reduction in the treatment of craniovertebral deformities.

**Objective:**

This study aims to describe our approach to individualized computer-simulated reduction and the design of C1-2 intra-articular 3D printed porous titanium alloy cages for the quantitative correction of craniovertebral junction deformities.

**Methods:**

A retrospective analysis was conducted on patients with craniovertebral deformities treated at our institution using individualized 3D-printed porous titanium alloy cages. Preoperative CT data were used to construct models for 3D realignment simulations. Cage designs were tailored to the simulated joint morphology following computer-assisted realignment. Preoperative and postoperative parameters were statistically analyzed.

**Results:**

Fourteen patients were included in the study, with a total of 28 3D-printed porous titanium alloy cages implanted. There were no cases of C2 nerve root resection or vertebral artery injury. All patients experienced symptom relief and stable implant fixation achieved in all cases. No implant-related complications were reported.

**Conclusion:**

The use of individualized computer-simulated reduction and the design of C1-2 intra-articular 3D printed porous titanium alloy cage facilitates precise 3D realignment in patients with craniovertebral deformities, demonstrating effectiveness in symptom relief and stability.

## Introduction

Congenital craniovertebral deformities include conditions such as basilar invagination (BI), atlantoaxial instability (AAI), atlas occipitalization, and Os odontoideum, among others [1]. These deformities are often characterized by atypical alignment of the bony structures at the craniovertebral junction (CVJ), where a misaligned odontoid process can exert pressure on the medulla oblongata, resulting in the gradual neurological deterioration [2].

The primary anomaly noted that can be addressed with intra-articular cage is the vertical displacement of BI. In this regard, Goel was the first to demonstrate the atlantoaxial intra-articular spacers [3]. However, congenital osseous abnormalities at the CVJ are often associated with three-dimensional (3D) deformities, including C1-2 rotational deformity, craniocervical kyphosis, C1 lateral inclination, and other issues. Therefore, restoring 3D balance is crucial for achieving complete realignment. The spacers described by Goel were relatively simple [3], making precise quantitative reduction challenging.

Recently, 3D printing technology has gained widespread use in spine surgery, providing surgeons with the ability to independently design prostheses [4]. This capability offers an ideal solution for achieving 3D quantitative realignment in patients with congenital craniovertebral deformities. This study describes our approach to preoperative computer-simulated reduction and C1-2 intra-articular cage design, as well as the effects of quantitative realignment.

## Method

### Patient population

We performed a retrospective analysis of patients with craniovertebral deformities who underwent treatment using 3D printed porous titanium alloy cages. All procedures were conducted by a single surgeon at our institution. All patients consented to the procedure. Patients’ osseous deformities are detailed in Table 1. The most common presenting symptom was limb weakness.

**Table 1:** Demographics

Case No.	Oseous deformity	Initial symptom
1	C2-3 fusion, AOZ, BI, AAI	Dizziness
2	C2-3 fusion, AOZ, BI, AAI	Limbs numbness
3	AOZ, BI, AAI	Hypaesthesia
4	AOZ, BI, AAI	Limbs numbness
5	BI, AAI	Gait disturbance
6	AOZ, BI, AAI	Headache
7	BI	Limb numbness
8	BI	Headache
9	BI	Neck pain
10	AOZ, BI, AAI, C2-3 fusion	Headache and dizziness
11	AOZ, BI, AAI, C2-3 fusion	Limb numbness and weakness
12	AOZ, BI, AAI	Limb weakness and gait disturbance
13	AOZ, BI, AAI, C2-3 fusion	Limb weakness
14	AAI, Os odontoideum	Limb weakness, torticollis

AOZ: atlas occipitalization; BI: basilar invagination; AAI: atlantoaxial instability

Patients who underwent fusion with polyether ether ketone cages, autologous bone grafting, or decompression without fusion were excluded from this study. Addition exclusions included patients with infection, tumors, trauma, or other related factors.

### Radiological evaluation

**Fig. 1:**
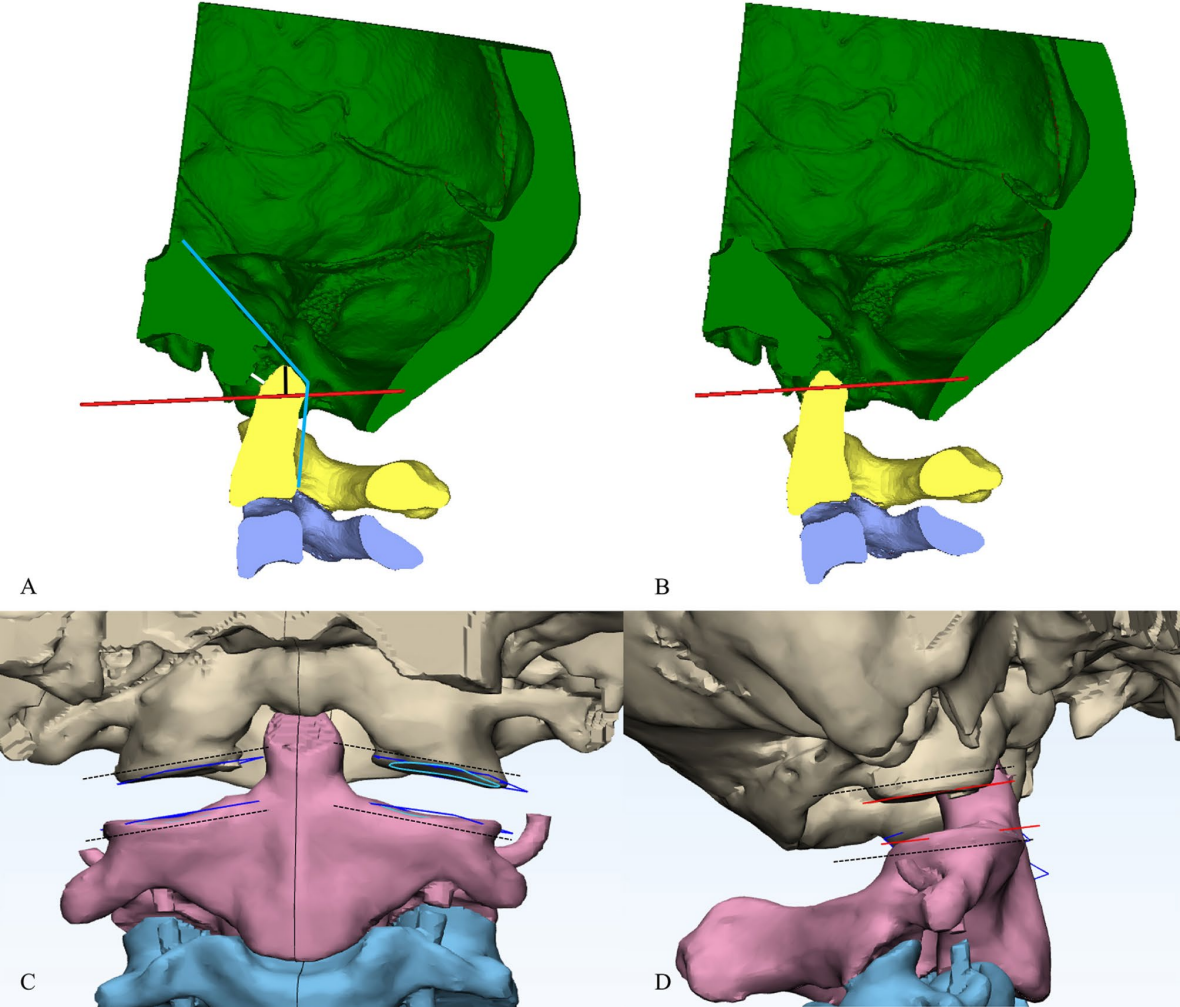
A schematic representation of bone reconstruction and computer-assisted sagittal realignment. (**A**) The midsagittal image showed the BI-AAI with atlas occipitalization. The red line represents the Chamberlain line. The black line represented the Chamberlain line violation. The angle formed by the posterior edge of clivus and the C2 vertebral body (blue lines) represented clivo-axial angle. The white line represented the atlantodental interval. (**B**) Through computer simulation, the odontoid process was moved caudally, resulting in a reduction of the Chamberlain line violation, atlantodental interval and an increase in the clivo-axial angle. (**C**) Due to the congruent relationship between the articular surfaces of facet joint, when viewed from the front, the upper and lower articular surfaces appear parallel. (**D**) As a result of adjusting the clivo-axial angle, there is an inclination angle between the upper and lower articular surfaces when viewed from the side

**Fig. 2:**
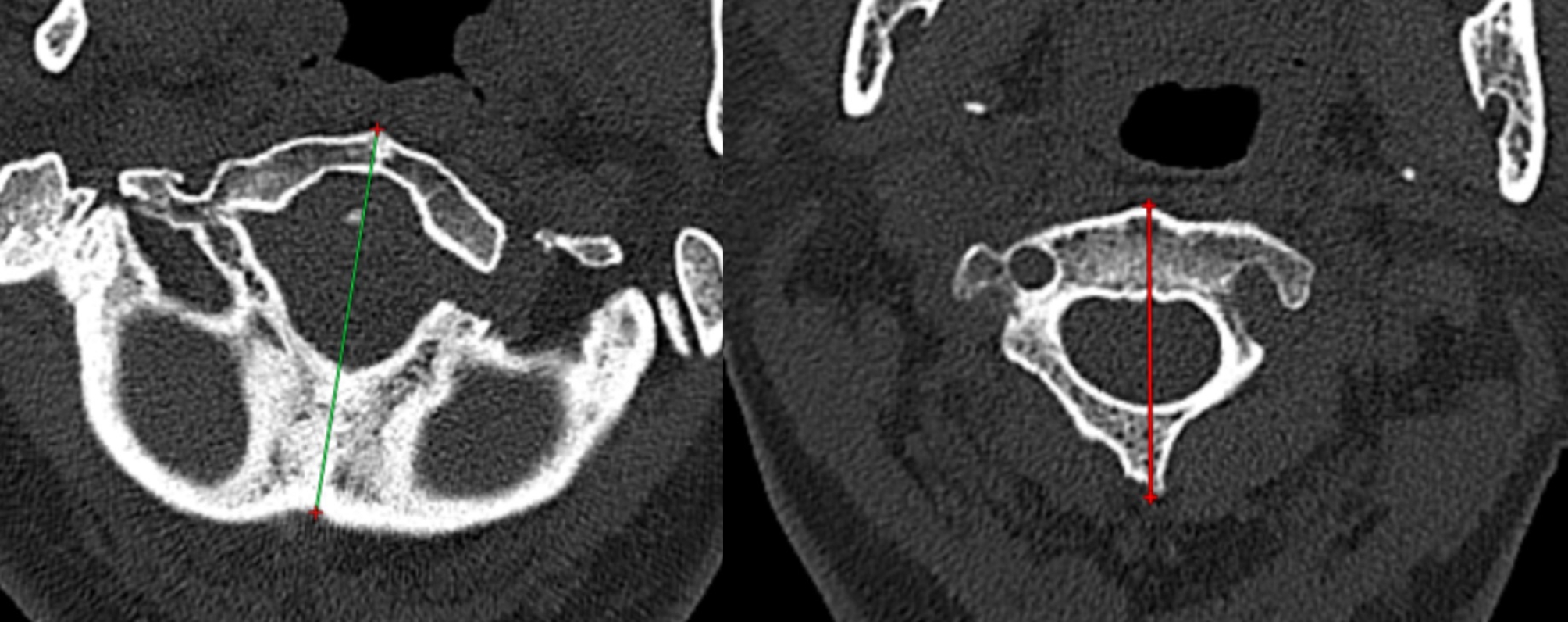
A schematic representation of C1-2 rotational angle. (**A**) A midline bisecting the C1 vertebra. C1 assimilation is present. (**B**) A midline bisecting the C2 vertebra. The angle between them represents the C1-2 rotational angle

**Fig. 3:**
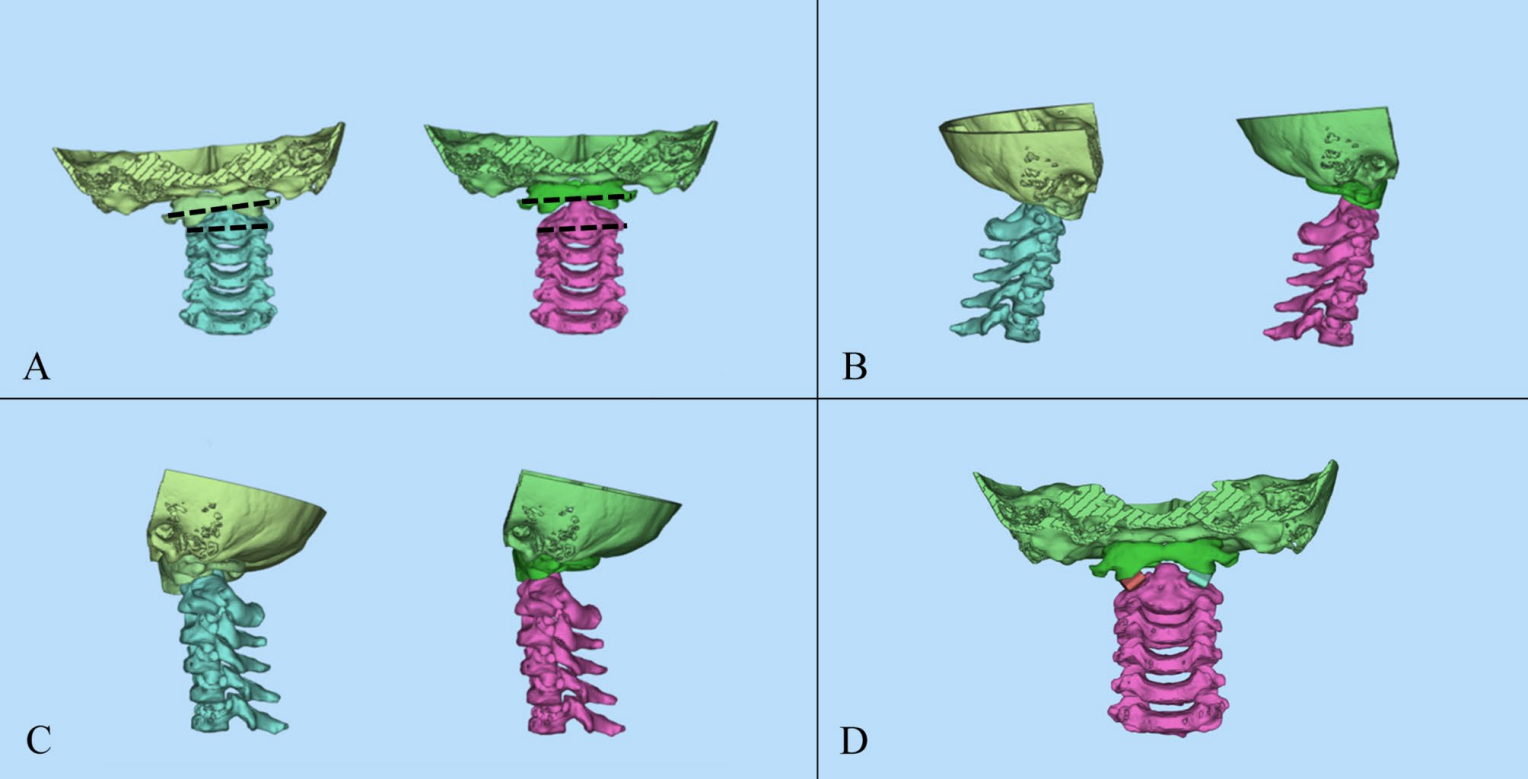
A schematic representation of 3D realignment. (**A**) Front view. After the adjustment on the midsagittal view, the 3D realignment was performed. The coronal balance was achieved. The C1 lateral inclination angle is delineated by the two dashed lines. (**B**) Right view. (**C**) Left view. (**D**) The cage was designed to be in direct contact with the upper and lower bony facet surfaces

Atlantodental interval (ADI), Chamberlain’s line violation (CLV), and clivo-axial angle (CXA) were measured on mid-sagittal CT images (Fig. 1). The C1-2 rotation angle was measured on axial CT images (Fig. 2), while the C1 lateral inclination angle was assessed on 3D models following Ishii et al. [5] (Fig. 3). The cervicomedullary angle (CMA) was measured on mid-sagittal MRI.

### Computer simulated reduction

#### 3D bone reconstruction

Head and neck CTA data were imported into Mimics software (Version 13.1, Materialise, Leuven, Belgium). The intelligent segmentation function was employed to delineate the upper cervical spine and skull for 3D models reconstruction.

#### Bone model realignment

The reposition function was used to stabilize the atlas and occiput while realigning the axis. The realignment criteria included achieving an ADI of < 1.5 mm, CLV of < 5 mm, and an increase in CXA by approximately 0°~12° (Fig. 1), while maintaining coronal balance. Coronal correction was performed by adjusting the C1 lateral inclination angle to 0° (Fig. 3). Correction of atlantoaxial rotational deformity was achieved by rotating the axis vertebra for alignment.

CXA correction was based on preoperative CXA and subaxial cervical spine lordosis. For patients with a CXA of < 145°, surgical correction is indicated, whereas minimal correction is required for those with a normal CXA. For patients with a significantly reduced CXA, a greater degree of correction is applied. Typically, cervical curvature within the range of -4° to 4° is considered straight, while curvature exceeding 4° is classified as kyphotic [6]. Postoperative outcomes showing either a straight or kyphotic cervical spine are undesirable. Thus, subaxial cervical lordosis falling below − 4° postoperatively is defined as unacceptable. To predict and mitigate development of postoperative subaxial cervical kyphosis, we adopted the methodology described by Liu et al. in which the change in CXA (ΔCXA) directly correlates with the change in subaxial cervical lordosis (Δ cervical curvature) [7]. Therefore, during surgery, ΔCXA should remain within the limits of the preoperative cervical curvature minus 4° to maintain an optimal postoperative curvature range and avoid creating a straight spine.

#### Cage design

Spacer designs were based on realigned bone models after computer simulations. The model was imported into 3-matic software for further design refinement. After outlining the atlantoaxial articular surface using the curve tool, the articular surface planes were obtained. Considering the C1-2 articular surfaces typically complement each other, only the inclination relationship correcting the CXA was obtained, as seen from a lateral view (Fig. 1).

**Fig. 4:**
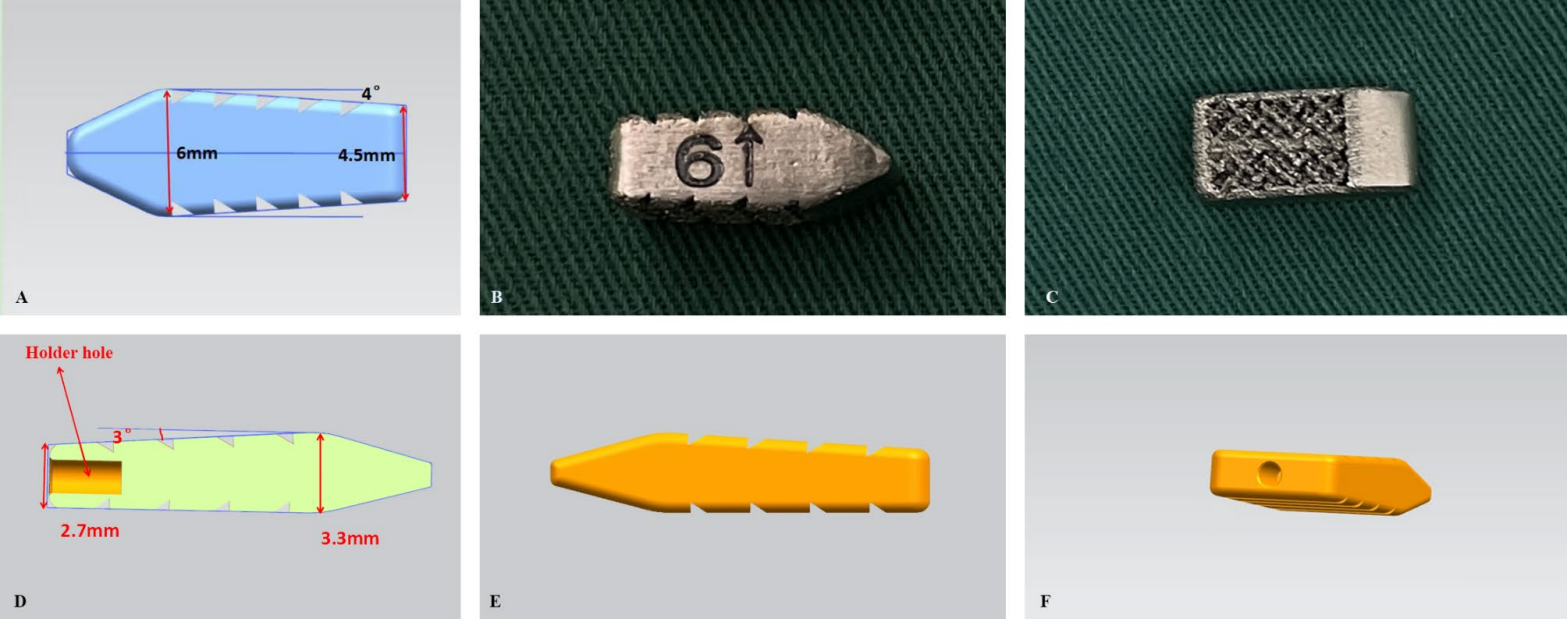
3D-printed porous titanium cage. (**A**) A schematic representation of a 6 mm height cage with an 8° inclination angle. (**B**) An actual image in profile view. (**C**) An overhead view. The anterior section of the cage is designed with a bullet-shaped, smooth surface to facilitate easy insertion between the joints. The posterior section serves as the fusion area, characterized by a porous titanium structure and a textured surface to enhance friction. (**D**) A schematic representation of a section view of a 3 mm height cage with a 6° inclination angle. (**E, F**) 3D schematics

Cages were designed to directly contact the upper and lower bony facet surfaces after realignment (Fig. 3). However, considering the fusion area between the articular surfaces and simplification of implantation during operation, a width of 8 mm was chosen for ease of insertion while ensuring sufficient fusion area. Coronal imbalance was corrected using cages of varying heights. The adjustable parameters included height and inclination angle, while width and length remained constant (Fig. 4).

The porous structure consisted of regular dodecahedral units with a porosity of 50-80%, a wire diameter of (550 ± 200) µm, and a pore size of (800 ± 200) µm (Fig. 4). The 3D data files of the cage after design confirmation were obtained. The cages were printed using Arcam EBM Q10 with Ti6A14VELI powder meeting ASTM F300l standards [4].

### Operation

Following general anesthesia, the patient was positioned prone, and a sliding-traction head frame was used [8]. A midline incision exposed the craniocervical junction from the occiput to C2, and laterally to the facets.

Bilateral C2 pars screws were inserted, with alternatives such as subfacetal screws or translaminar screws if there was a high-riding vertebral artery [9]. The C2 nerve roots were gently elevated using a micro dissector, and ultrasonic osteotomy was employed for joint cartilage removal. After releasing both joints, traction was gradually increased to 1/6 of the patient’s body weight. At this stage, the joint space gradually expanded.

A distractor was placed into the joint space, followed by the bilateral implantation of the 3D-printed cages. Two titanium rods were connected to secure the occiput and cervical spine. Horizontal realignment was achieved using the cantilever technique. The surgical procedure involves pre-bending titanium rods to the appropriate curvature based on the preoperative simulated correction required for the CXA. The rods were first secured at the C2 end, while leaving a gap between the cranial end of the rod and the occiput. Anterior displacement of the axis vertebra and CXA correction were then achieved by pushing the cranial end of the titanium rod towards the occiput. In patients without atlas occipitalization, the fixation segment was limited to the C1-2 segment. Horizontal realignment of the axis vertebra was achieved by pre-bending the titanium rod and gradually securing them to the C1 lateral mass screw, completing the realignment process.

### Follow-up

Patients were followed up at 3 and 12 months postoperatively. MRI and CT scans were used to evaluate neural and osseous structures, respectively. Due to the porous nature of the cages, bone growth within the cages cannot be observed radiologically; therefore, implant stability was assessed using CT. Implant stability is determined by the absence of implant displacement or loosening.

### Statistics

Continuous variables were expressed as mean ± standard deviation. Preoperative and postoperative comparisons were performed using a paired t-test, with statistical significance defined as *P* < 0.05.

## Results

### Participants and operation

Fourteen patients (5 males, 9 females; mean age: 46.07 ± 12.80 years) were included. Thirteen patients had BI, eleven had AAI, and one had Os odontoideum.

A total of 28 3D-printed porous titanium alloy cages were implanted, with no cases of C2 nerve root resection, vertebral artery or nerve injury, or cerebrospinal fluid leak. One patient developed postoperative pneumonia which resolved with antibiotics. Another patient experienced fat liquefaction at the incision site, requiring debridement.

### Cage design

The cage featured a bullet-shaped anterior part with a smooth surface for easy insertion and a rough, porous posterior part to enhance friction, prevent displacement, and promote fusion (Fig. 4). Heights ranged from 3 to 9 mm, with six options: 3 mm, 5 mm, 6 mm, 7 mm, 8 mm, and 9 mm. Inclination angles ranged from 6 to 12°, with four options: 6°, 8°, 10°, and 12°.

### Reduction effect

Preoperative CLV was 7.39 ± 3.90 mm, improving to 2.82 ± 3.42 mm postoperatively. Preoperative ADI was 3.61 ± 2.51 mm, improving to 1.40 ± 0.92 mm. Preoperative CXA was 138.70 ± 13.91°, improving to 145.24 ± 7.93°. Preoperative CMA was 137.11 ± 13.39°, improving to 152.63 ± 6.25°. Preoperative lateral inclination angle was 4.09 ± 3.76°, improving to 1.06 ± 0.98°. Preoperative rotational angle was 6.83 ± 5.48°, improving to 2.22 ± 1.72°. All preoperative to follow-up changes were statistically significant (Table 2).

**Table 2:** Preoperative and the latest follow-up parameters

	CLV	ADI	CXA	CMA	Lateral inclination angle	Rotational angle	JOA score	VAS
Preoperative	7.39 ± 3.90	3.61 ± 2.51	138.70 ± 13.91	137.11 ± 13.39	4.09 ± 3.76	6.83 ± 5.48	12.21 ± 1.93	3.2 ± 0.8
Postoperative	2.82 ± 3.42	1.40 ± 0.92	145.24 ± 7.93	152.63 ± 6.25	1.06 ± 0.98	2.22 ± 1.72	14.14 ± 2.07	1.0 ± 0.6
p	< 0.001	0.003	0.028	< 0.001	0.004	0.002	< 0.001	< 0.001

CMA: cervicomedullary angle

JOA: Japanese Orthopaedic Association

### Follow-up

**Fig. 5:**
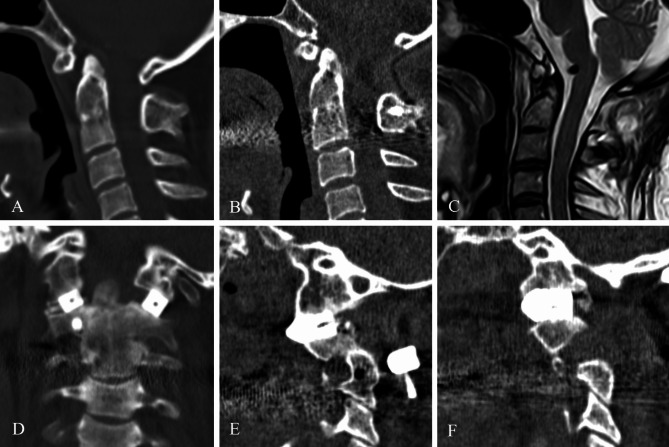
Illustrative case. A 50-year-old female presented with limbs weakness. (**A**) Preoperative sagittal CT showed BI-AAI with atlas occipitalization and congenital C2-3 fusion. (**B**) The latest-follow-up sagittal CT showed good reduction. (**C**) The latest-follow-up MRI showed the relieved foramen magnum stenosis. (**D**) Coronal CT showed good position of 3D-printed porous titanium cages. (**E, F**) parasagittal CT showed stable implants

The average follow up was 13.7 ± 6.2 months. All patients showed symptom relief, with no neurological decline. JOA score improved from 12.21 ± 1.93 preoperatively to 14.14 ± 2.07 (*P* < 0.001). All implants were stable (Fig. 5), with no implant-related complications. Six patients had pain before the surgery. VAS score improved from 3.2 ± 0.8 preoperatively to 1.0 ± 0.6 (*P* < 0.001).

## Discussion

The treatment of congenital craniovertebral deformities has evolved from decompression alone to reduction and fixation of the atlantoaxial vertebrae. Early reduction and fixation primarily aim to alleviate the compression of the medulla oblongata but often neglect the normal curvature and mechanical transmission of the CVJ, leading to suboptimal outcomes including worsened craniocervical kyphosis [10], inadequate vertical realignment, and residual coronal imbalance. Goel et al. proposed that instability is the primary cause of these deformities, advocating standalone fusion procedure without spacers as the optimal treatment [11, 12]. However, such approaches may not fully correct the complex 3D misalignment commonly associated with these deformities. As a result, it may fail to adequately address the foramen magnum stenosis and neural elements compression.

Cage implantation offers a promising solution for achieving better realignment. Earlier designs by Goel were rudimentary, with limited height (3 mm), falling short of achieving sufficient vertical realignment. Later developments such as autogenous bone or anterior cervical discectomy and fusion cages reported for C1-2 intra-articular fusion [13–15] also faced limitations in their adaptability, often requiring intraoperative trimming [14].

Recent advancements have enabled better reduction rates and spurred research into quantitative reduction methods. In 2016, Salunke introduced a treatment approach for BI-AAI with coronal imbalance. This involved the implantation of cages of variable heights on different sides to correct the coronal imbalance [16]. In 2020, Guan et al. developed cages with sagittal inclinations for CXA correction [17]. However, these techniques have largely focused on adjusting the CXA, neglecting the comprehensive 3D aspects of craniovertebral deformities such as atlas occipitalization and C2-3 fusion, and atlas shrug and rotational deformity. A comprehensive reduction strategy for CVJ deformities should consider 3D aspects, including the reduction of BI-AAI, craniovertebral junction kyphosis, rotational deformity, and coronal imbalance. Previous quantitative reduction techniques have predominantly focused on sagittal correction, with limited attention to achieving true 3D reduction.

In this study, we employed advanced medical modeling software for 3D preoperative planning and computer-assisted reduction to achieve quantitative 3D alignment. Although it is challenging to precisely control the degree of interfacet distraction during surgery, we accomplished individualized 3D quantitative reduction through careful preoperative planning of bilateral cage placement to reshape and correct the articular surface. Preoperative adjustments of the CXA were integrated into the intraoperative pre-bending of rods, enhancing the reduction effect. Postoperative outcomes were satisfactory, demonstrating that individualized computer-simulated preoperative reduction with 3D-printed titanium cages provides a viable method for 3D quantitative correction.

Key technical considerations include: (1) Avoiding the design of excessively tall cages in patients with severe CLV. In cases where tension bands restrict intraoperative intra-articular distraction, cage placement may be limited, especially in patients with BI and no AAD. Excessive distraction may compromise neural structures, making it unrealistic to achieve a CLV of less than 5 mm in such patients. A combination of joint release and distraction, and skull traction is often effective, in reducing deformities but comes with inherent limitations. Therefore, our approach did not apply these standards for patients with significant CLV, who were excluded from this study. Preoperative simulations of vertical reduction should be cautiously interpreted and not overestimated. (2) Careful correction of craniocervical kyphosis and subaxial hyperflexion deformities is crucial to avoid postoperative subaxial cervical kyphosis [18]. (3) In patients with articular surface mismatch after computer-simulated reduction, zero-profile cages should be designed to prevent anterior soft tissue compression. (4) Skull traction is essential for intraoperative reduction. In this study, C1-C2 distraction using a sliding-traction head frame preserved articular surface integrity [8], avoiding cage mismatch due to articular surface damage and ensuring successful 3D quantitative reduction.

The advantages of this technique include: (1) The achievement of 3D quantitative reduction using individualized 3D-printed porous titanium alloy cages. (2) Unlike traditional methods, the use of these cages obviates the need for bone grafting, thus reducing the risk of complications associated with iliac bone harvesting. (3) The porous titanium alloy cages offer enhanced friction, reducing the likelihood of cage displacement. (4) These cages possess an appropriate elastic modulus and extensive contact area, minimizing the risk of implant subsidence.

However, this technique has limitations. First, joint distraction has inherent limitations, and the preoperative goal of achieving a CLV of less than 5 mm is not applicable to all patients. In cases of severe CLV, achieving this standard may be unfeasible. For mild CLV cases, a combination of joint release, gradual distraction, and traction can effectively reduce CLV to below 5 mm postoperatively. The highest CLV in this study was 11.2 mm. Additionally, this study is based on bony structure simulations; future approaches should incorporate neural structure simulations to better evaluate nerve morphology pre- and postoperatively. Second, variations in the vertebral artery may increase the risk of injury during posterior intra-articular manipulation. Third, the joint release using ultrasonic bone scalpel may damage the articular surfaces, potentially causing cage subsidence, which underscores the importance of minimizing unnecessary resection of the articular surface [19]. Fourth, the high cost and lengthy production process of 3D-printed implants are notable drawbacks. Furthermore, direct imaging assessment of fusion in 3D-printed cages is limited; indirect signs, such as implant stability and bone integration, are relied upon, necessitating regular follow-ups. Unlike traditional bone grafts, direct signs, such as bridging trabeculae across the fusion site or the absence of continuous radiolucent lines or areas in that area, are not visible on CT [20]. Finally, the study’s small sample size limits the generalizability of its finding, warranting further validation through larger case series.

## Conclusion

Individualized computer-simulated reduction and the use of C1-2 intra-articular 3D printed porous titanium alloy cages contribute to effective 3D realignment in patients with craniovertebral deformities. The development of customized 3D-printed atlantoaxial cages offers a promising approach for addressing complex CVJ deformities.

## Data Availability

No datasets were generated or analysed during the current study.

## Declarations

### Presentation

The manuscript has been read and approved by all the authors. This manuscript has not been published or presented elsewhere in part or in entirety and is not under consideration by another journal.
